# Abnormal subcellular localization of GABA_A_ receptor subunits in schizophrenia brain

**DOI:** 10.1038/tp.2015.102

**Published:** 2015-08-04

**Authors:** T M Mueller, C E Remedies, V Haroutunian, J H Meador-Woodruff

**Affiliations:** 1 Department of Psychiatry and Behavioral Neurobiology, University of Alabama at Birmingham, Birmingham, AL, USA; 2 Science and Technology Honors Program, University of Alabama at Birmingham, Birmingham, AL, USA; 3 Department of Psychiatry, Mount Sinai School of Medicine, New York, NY, USA

## Abstract

Inhibitory neurotransmission is primarily mediated by γ-aminobutyric acid (GABA) activating synaptic GABA type A receptors (GABA_A_R). In schizophrenia, presynaptic GABAergic signaling deficits are among the most replicated findings; however, postsynaptic GABAergic deficits are less well characterized. Our lab has previously demonstrated that although there is no difference in total protein expression of the α1–6, β1–3 or γ2 GABA_A_R subunits in the superior temporal gyrus (STG) in schizophrenia, the α1, β1 and β2 GABA_A_R subunits are abnormally *N*-glycosylated. *N*-glycosylation is a posttranslational modification that has important functional roles in protein folding, multimer assembly and forward trafficking. To investigate the impact that altered *N*-glycosylation has on the assembly and trafficking of GABA_A_Rs in schizophrenia, this study used western blot analysis to measure the expression of α1, α2, β1, β2 and γ2 GABA_A_R subunits in subcellular fractions enriched for endoplasmic reticulum (ER) and synapses (SYN) from STG of schizophrenia (*N*=16) and comparison (*N*=14) subjects and found evidence of abnormal localization of the β1 and β2 GABA_A_R subunits and subunit isoforms in schizophrenia. The β2 subunit is expressed as three isoforms at 52 kDa (β2_52 kDa_), 50 kDa (β2_50 kDa_) and 48 kDa (β2_48 kDa_). In the ER, we found increased total β2 GABA_A_R subunit (β2_ALL_) expression driven by increased β2_50 kDa_, a decreased ratio of β2_48 kDa_:β2_ALL_ and an increased ratio of β2_50 kDa_:β2_48 kDa_. Decreased ratios of β1:β2_ALL_ and β1:β2_50 kDa_ in both the ER and SYN fractions and an increased ratio of β2_52 kDa_:β2_48 kDa_ at the synapse were also identified in schizophrenia. Taken together, these findings provide evidence that alterations of *N*-glycosylation may contribute to GABAergic signaling deficits in schizophrenia by disrupting the assembly and trafficking of GABA_A_Rs.

## Introduction

Schizophrenia is a chronic psychiatric disorder that affects multiple brain regions, neurotransmitter systems and cell types, and presents with variable combinations of symptoms. Negative and cognitive symptoms associated with this illness have a profound effect on patient outcome, and have been shown to correlate with dysfunctional GABAergic signaling.^1, 2, 3, 4, 5, 6, 7, 8, 9, 10, 11, 12^ A consistent finding in schizophrenia research is the decreased expression of GAD67, an enzyme necessary for the synthesis of the neurotransmitter γ-aminobutyric acid (GABA).^13, 14, 15, 16, 17, 18, 19, 20, 21^ Altered inhibitory neurotransmission from GABAergic interneurons onto cortical pyramidal neurons has been shown to disrupt the excitatory:inhibitory balance in the cortex and contribute to disruptions of neural synchrony in schizophrenia^1, 3, 5, 6, 9, 22, 23^ and other neuropsychiatric disorders.^5, 6^ These presynaptic GABAergic deficits have been extensively studied in schizophrenia, whereas postsynaptic GABA_A_ receptor (GABA_A_R) subunit abnormalities have been more difficult to characterize due to extensive homology between subunits and the variety of potential subunits that may be expressed and incorporated into intact receptors.^6, 24, 25, 26, 27^

Alterations of transcript and protein expression of several GABA_A_R subunits in a brain-region, cortical lamina and cell type-specific manner have been described in schizophrenia.^13, 15, 17, 28, 29, 30, 31, 32, 33, 34, 35^ We have previously reported that there is no change to the total protein expression of the α1–6, β1–3 and γ2 GABA_A_R subunits in the superior temporal gyrus (STG; Brodmann area 22),^36^ an area that we focused on given prior studies indicating decreased volume, increased GABA_A_R density and GABAergic signaling abnormalities in this cortical region in schizophrenia.^37, 38, 39, 40^ Although protein expression of these GABA_A_R subunits was unchanged in STG in schizophrenia, we identified significant alterations in the posttranslational processing of the α1, β1 and β2 GABA_A_R subunits; specifically, we observed abnormalities of immature *N*-linked glycosylation of the α1 and β1 GABA_A_R subunits, and altered total *N*-glycosylation of the β2 GABA_A_R subunit in schizophrenia.^36^

*N*-glycosylation has an essential role in proper protein folding and assembly, endoplasmic reticulum (ER) quality control mechanisms and forward trafficking from the ER to the plasma membrane.^41, 42, 43, 44, 45, 46, 47, 48^ Previous studies suggested that these functional processes are disrupted in schizophrenia, and we have identified alterations of *N*-linked glycosylation of multiple neurotransmitter-associated proteins that are consistent with abnormal ER function.^36, 49, 50, 51^ A smaller immature *N*-glycan has been observed attached to the α1 GABA_A_R subunit in schizophrenia, which suggests that this subunit undergoes early glycoprotein processing and may be retained in the calnexin–calreticulin protein folding cycle in the ER. More of the β1_49 kDa_ GABA_A_R subunit isoform is immaturely glycosylated in schizophrenia, which could result in increased incorporation of this subunit into synaptically targeted GABA_A_Rs. The abnormal total *N*-glycosylation of the β2 subunit that we have previously reported may alter receptor targeting after ER exit and result in decreased β2 expression at the synapse. As *N*-glycosylation abnormalities are evident on isoforms of both the β1 and β2 GABA_A_R subunits, this atypical pattern of posttranslational modifications may serve to ensure preferential incorporation of one β-subunit over the other to compensate for presynaptic GABAergic signaling deficits in the disorder. Accordingly, we predict that the ratio of β1-containing versus β2-containing GABA_A_Rs could be altered at the synapse. On the basis of the previously reported *N*-glycosylation deficits in schizophrenia, we hypothesize that deficits in initial protein processing, including abnormal posttranslational protein modifications, may alter neurotransmitter receptor assembly and trafficking and contribute to the pathophysiology of schizophrenia.

To ascertain whether *N*-glycosylation alterations contribute to aberrant forward trafficking of GABA_A_Rs in schizophrenia, in this study, we examined the subcellular localization of GABA_A_R subunits that we previously found to be abnormally *N*-glycosylated in STG in schizophrenia. We also examined the subcellular distribution of the γ2 GABA_A_R subunit given its role in synaptic targeting of intact receptors,^52, 53, 54, 55, 56, 57, 58^ and the α2 GABA_A_R subunit, which has been implicated in schizophrenia.^32, 59, 60, 61^ We determined the expression of the α1, α2, β1, β2 and γ2 GABA_A_R subunits in defined subcellular compartments from postmortem STG from schizophrenia and comparison subjects. By assessing the abundance of these GABA_A_R subunits in subcellular compartments at the proximal and distal ends of the forward trafficking pathway, we anticipated that we would identify alterations in subcellular localization or subunit composition of intact GABA_A_Rs in schizophrenia that may contribute to the pathophysiology of this disorder.

## Materials and methods

### Subjects and tissue acquisition

Samples of the full thickness of gray matter from the left STG (Brodmann area 22) of 16 schizophrenia subjects and 14 non-psychiatrically ill comparison subjects (Table 1 and Supplementary Table S1) were obtained from the Mount Sinai Medical Center brain collection, as previously described.^36, 62, 63^ Patients who were diagnosed with schizophrenia on the basis of DSM-III-R criteria and confirmed by at least two clinicians, had a documented history of the onset of psychotic symptoms before age 40 and at least 10 years of hospitalization, were prospectively recruited.^64^ Each subject was assessed for psychiatric illnesses, history of drug or alcohol abuse and tests of cognition. CERAD guidelines were used to evaluate the brain macro- and microscopically.^65^ Comparison subjects were similarly evaluated, and had no history of documented substance abuse or psychiatric illness. Subjects that had been in a coma for more than 6 h before death, had a history of substance abuse or death as a result of suicide were excluded from this study. Consent to perform an autopsy on the body and brain for diagnostic and research purposes was obtained from the next-of-kin for each subject.^63, 65^ Tissue samples were pulverized with small amounts of liquid nitrogen and stored at −80 °C until processed for study.

### Subcellular fractionation

**Figure 1:**
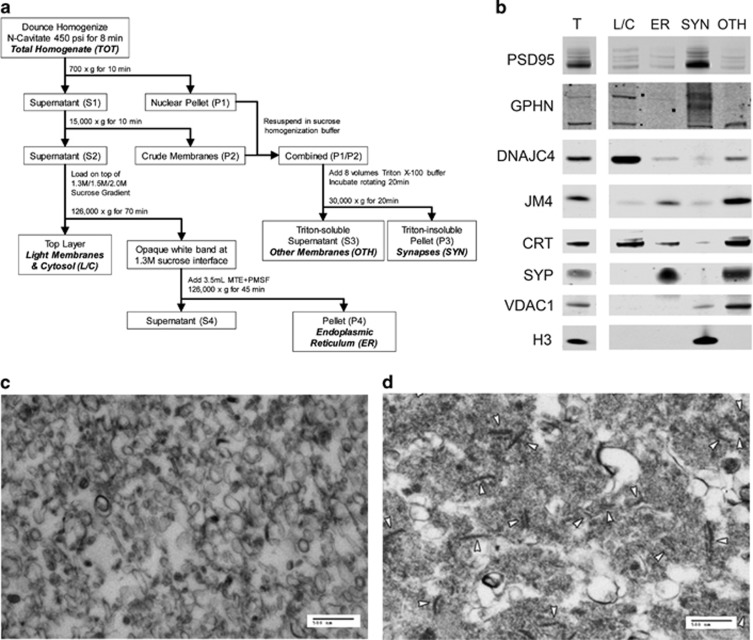
Fractions enriched for light membranes and cytosol (L/C), endoplasmic reticulum (ER), excitatory and inhibitory synapses (SYN) and other intermediate membranes (OTH) generated from postmortem human cortex. (**a**) Schematic depiction of the centrifugation, differential sucrose gradient and Triton solubilization steps to produce L/C, ER, SYN and OTH-enriched fractions from nitrogen-cavitated samples. Briefly, after nitrogen-cavitated cortical homogenate samples undergo sequential centrifugations, S2 is loaded on top of a differential sucrose gradient and ultracentrifuged to separate the ER from other light membranes and cytosolic components based on membrane density. The ER membranes appear as a semi-opaque white band, and the L/C remains suspended in the translucent top layer. P1 and P2 from the first centrifugation steps are resuspended, combined and solubilized by a brief incubation with Triton X-100 buffer. Following centrifugation, the Triton-insoluble synaptic membranes are concentrated in the resulting P3, while S3 contains the remaining heavy and intermediate membrane components. (**b**) Representative images from western blots of total homogenate, L/C, ER, SYN and OTH fractions probed for various subcellular markers to validate the efficacy of the fractionation method in postmortem human cortex. Target marker proteins include postsynaptic density protein 95 (PSD95), for excitatory synapses; gephyrin (GPHN), for inhibitory synapses and extrasynaptic membrane; DnaJ/hsp40 homolog subfamily C member 4 (DNAJC4), for cytosol; PRA1 family protein 2 (JM4), for ER and Golgi membranes; calreticulin (CRT), for ER lumen; synaptophysin (SYP), for extrasynaptic membranes; voltage dependent anion-selective channel protein 1 (VDAC), for mitochondria; and histone 3 (H3), for nuclei. (**c** and **d**) Representative electron microscopy (EM) image of the ER and SYN fractions (scale bars, 500 nm). (**c**) ER membrane is enriched and no other identifiable structures or organelles are evident in the ER fraction. (**d**) Synapses, indicated by white arrowheads, are enriched and no other intact structures or organelles are visualized in the SYN fraction. MTE, D-mannitol, Tris-base, and EDTA; PMSF, phenylmethylsulfonyl fluoride.

Subcellular fractionation was performed using nitrogen cavitation, differential sucrose gradient ultracentrifugation and Triton solubilization (Figure 1a). This protocol yields fractions enriched for light membrane/cytosol, ER, and synapses (SYN). It also yields a relatively nonspecific residual fraction containing markers for mitochondria, extrasynaptic membranes, ER lumen and other membrane and vesicle-associated proteins; this triton-soluble fraction, referred to as the ‘other intermediate membrane' fraction, does not contain nuclear or excitatory synaptic markers (Figure 1b).

For each subject, 50 mg of pulverized tissue was homogenized on ice by 10 strokes in a glass–teflon homogenizer in 1.25 ml of 1 × Isotonic Extraction Buffer (Sigma-Aldrich, St. Louis, MO, USA) diluted with sterile water, then transferred into a nitrogen cavitation vessel (Parr Instrument Company, Moline, IL, USA) and pressurized at 450 psi for 8 min for further disruption of cell membranes.^66, 67^ The homogenates were collected through the outlet port of the vessel by nitrogen decompression; 950 μl was used for subcellular fractionation and the remainder reserved as total homogenate.

The homogenate from each subject was centrifuged at 700 *g* for 10 min at 4 °C. The supernatant (S1) was subsequently centrifuged at 15 000 *g* for 10 min at 4 °C and the pellet (P1) was resuspended in 75 μl of sucrose homogenization buffer (5 mM Tris-HCl, pH 7.4, 320 mM sucrose and a protease inhibitor tablet (Complete Mini; Roche Diagnostics, Mannheim, Germany)). After the second centrifugation, the supernatant (S2) was loaded on top of a differential sucrose gradient (prepared with 1 ml each of 2.0 M sucrose, 1.5 M sucrose, then 1.3 M sucrose in a 14 × 89 mm polyallomer ultracentrifuge tube (Beckman Coulter, Indianapolis, IN, USA)), and the pellet (P2) was resuspended in 75 μl sucrose homogenization buffer and combined with the resuspended P1.^66, 67^

To the combined P1/P2 resuspension, 1.2 ml of Triton X-100 buffer (10 mM Tris-HCl, pH 7.4, 1 mM Na_3_VO_4_, 5 mM NaF, 1 mM EDTA, 1 mM EGTA, 5%v/v Triton X-100) was added and samples were incubated for 20 min at 4 °C on a rotator before being centrifuged for 20 min at 30 000 *g* at 4 °C. The triton-insoluble pellet was resuspended in 125–150 μl of 1 × phosphate-buffered saline (PBS) with a protease inhibitor tablet (Roche Diagnostics) and sonicated 5 × for 1 s at level 4 (Sonic Dismembrator Model 100, Fisher Scientific, Pittsburgh, PA, USA) to produce the final SYN fraction.^68^ The supernatant (S3) was reserved to produce the final other intermediate membrane fraction.^68^

The sucrose gradient was ultracentrifuged at 126 000 *g* (35 000 r.p.m. in a SW60Ti rotor (Beckman Coulter)) at 4 °C for 70 min. The upper layer was reserved to produce the final light membrane/cytosol fraction.^66, 67^ A dense, semi-opaque white band at the interface of the upper layer and the 1.3 M sucrose layer was aspirated and combined with 3.0–3.5 ml of ice-cold 1 × MTE+PMSF buffer (270 mM D-mannitol, 10 mM Tris-base and 0.1 mM EDTA adjusted to pH 7.4, with 1 mM phenylmethylsulfonyl fluoride) and ultracentrifuged in a new polyallomer ultracentrifuge tube at 126 000 *g* at 4 °C for 45 min. The supernatant was decanted and pellet dried for 2–3 min before being resuspended in 50 μl of ice-cold 1 × PBS with 0.5% v/v Triton X-100, pH 7.4, to produce the final ER fraction.^66, 67^

### Electron microscopy

To validate enrichment of ER membranes in the ER fraction (Figure 1c) and symmetrical and asymmetrical synapses in the SYN fraction (Figure 1d) by electron microscopy (EM), fraction samples from two non-psychiatrically ill subjects were prepared as previously described.^66, 67^ Briefly, fractions were fixed in 4% gluteraldehyde in 0.1 M cacodylate buffer (pH 7.4) at 4 °C for at least 24 h. The University of Alabama at Birmingham HRIF Electron Microscopy Core then processed the samples and post-stained with uranyl acetate and lead citrate for EM imaging on a Tecnai F20 FEG transmission electron microscope (FEI, Hillsboro, OR, USA).

### Western blot sample preparation

Protein concentration of the homogenate and fraction samples was determined with BCA assays (Thermo Fisher Scientific, Pittsburgh, PA, USA). Western blot samples were prepared by dilution with sucrose homogenization buffer and the addition of 6 × loading buffer (0.5 M Tris-HCl, 36% glycerol, 4.5% sodium dodecyl sulfate and 2% β-mercaptoethanol) to a final protein concentration of 0.556 μg μl^−1^ (10 μg in 18 μl).

### Deglycosylation

Peptide *N*-glycosidase F (PNGase F; New England Biolabs, Ipswich, MA, USA) was used to cleave total *N*-glycans in samples of total homogenate, ER and SYN fractions. For each fraction, 25 μg of protein was denatured with Denaturation Solution (New England Biolabs) and 10 × PNGase F Reaction Buffer (New England Biolabs) by incubation at 70 °C for 10 min. The deglycosylating enzyme PNGase F and 10% NP40 were added and samples incubated overnight at 40 °C. To each sample, 6 × loading buffer was added and heated at 70 °C for 10 min. Non-enzyme-treated negative control samples with or without NP40 were prepared identically to the enzyme-treated samples with the same buffers, replacing the enzyme and NP40 with water.^36, 49, 50, 51^

### Western blot analysis

Fraction samples were run on three 12-well 4–12% Bis-Tris polyacrylamide gels (Life Technologies, Grand Island, NY, USA). For each subject, 10 μg of total homogenate, light membrane/cytosol, ER, SYN and other intermediate membrane fractions were loaded. Novex Sharp Pre-stained Protein Standard (Life Technologies) was run on each gel. Gels were suspended in a bath of 1 × NuPAGE MES sodium dodecyl sulfate running buffer (Life Technologies) and run on a Novex Mini Cell nuPAGE system (Life Technologies) at 55 V for 20 min, followed by 150 V for 80 min.

After electrophoresis, proteins were transferred onto 0.45 μm nitrocellulose membranes (Bio-Rad, Hercules, CA, USA) at 16 V for 30 min using a Bio-Rad semi-dry transfer apparatus. Membranes were cut just above 160 kDa, just below 60 kDa, and just above 40 kDa, followed by a brief PBS rinse. For each set of three gels, membranes of the same molecular weight range were incubated using the appropriate primary antibody in the same box, except for the 60–40 kDa range membranes, which were probed separately for GABA_A_R subunits. Membranes were incubated with primary antibodies against VCP, gephryin, JM4, DNAJC4 and the α1, α2, β1, β2, γ2 GABA_A_R subunits (Supplementary Table S2). Conditions for primary antibodies were optimized to be within the linear range of detection for the Odyssey Infrared Imaging System (LI-COR Biosciences, Lincoln, NE, USA) at a resolution of 169 μm and intensity level of 3. After washing three times with 1 × PBS+0.1% Tween, each membrane was incubated with the appropriate IRDye-labeled secondary antibody (LI-COR Biosciences) for 1 h, then washed twice with PBS+0.1% Tween and once with MilliQ water before being scanned. After scanning, the membranes were stored in MilliQ water at 4 °C.

Antibody specificity for the GABA_A_R subunits was determined by comparison of the predicted molecular mass of the target protein with the antibody manufacturers observed molecular mass and observed molecular mass of immunoreactive bands from western blots of postmortem cortical homogenate from a non-psychiatrically ill comparison subject. The α1, α2 and γ2 GABA_A_R antibodies strongly associated with protein bands at the expected molecular mass of the respective subunit. Specificity of the GABA_A_R β1 subunit was determined by incubating the primary antibody with the antigenic peptide (sc-31426P, Santa Cruz Biotechnology, Santa Cruz, CA, USA) for 10 min before probing a western blot of total cortical homogenate. Protein bands, which were not evident after this incubation but were apparent when probed with primary antibody alone were determined to represent the GABA_A_R β1 subunit. There was no peptide antigen available for the GABA_A_R β2 subunit; however, similar to the GABA_A_R β1 subunit, we were able to verify which bands represented GABA_A_R β3 subunits using the antigenic peptide (sc-31430P, Santa Cruz Biotechnology) incubated with the GABA_A_R β3 antibody (sc-31430, Santa Cruz Biotechnology) and comparing western blots to identify which bands were specific to β3 subunit expression. We then compared these with a western blot probed with an antibody that recognizes all the three GABA_A_R β-subunits (sc-28794, Santa Cruz Biotechnology) and identified the immunoreactive bands that were recognized by the GABA_A_R β2 antibody, but not the β1 or β3 subunit antibodies, as representing the GABA_A_R β2 subunit.

### Data analysis

Image Studio software (LI-COR Biosciences) was used to collect the near-infrared fluorescence value, expressed as signal with left–right median intralane background subtracted, for each protein band under investigation in the total homogenate and subcellular fractions. The number of subjects per group was determined using the previously reported mean and standard deviation of GABA_A_R α1, β1 and β2 protein expression in STG to detect a 20% difference with statistical power=0.80. The protein expression of α1, α2, β1, β2 and γ2 GABA_A_R subunits, as well as the protein expression of gephyrin and JM4, was determined by measuring the signal intensity of each band and normalizing to a loading control as well as a marker for each specific fraction. We used VCP as the loading control due to its ubiquitous expression in brain, immunoreactivity in each subcellular fraction, and unchanged expression in multiple brain regions in schizophrenia.^36, 69, 70^ We used gephyrin as the normalizing factor for the SYN fraction because of its role as a cytoskeletal scaffold for GABA_A_R-containing synapses.^56, 58, 71, 72^ JM4 is a marker of ER and Golgi membranes expressed in cortical neurons used as the normalizing factor for the ER fraction due to its consistent and uniform expression in that fraction. Before GABA_A_R subunit normalization, we verified that VCP, gephyrin and JM4 were not different between groups in the total homogenate or subcellular fractions. For the α2 and β2 GABA_A_R subunits, which are expressed as multiple isoforms, individual protein bands for each isoform and all isoform bands together, were measured. Although the β1 GABA_A_R subunit is also expressed as a doublet in our western blot conditions, the individual isoforms did not have enough separation between bands to be measured individually.

VCP-normalized signal intensity was used to assess protein expression for each target in the total homogenate lanes to validate our prior finding of unchanged GABA_A_R subunit expression in schizophrenia STG from a different subject cohort^36^ and for use as a within-subject normalizing factor for protein expression in subcellular fractions. For the ER fraction lane, VCP-normalized GABA_A_R subunit signal intensity was normalized to VCP-normalized JM4 signal intensity and divided by the VCP-normalized GABA_A_R subunit signal intensity in the total homogenate lane. Similarly, for the SYN fraction lane, VCP-normalized GABA_A_R subunit signal intensity was normalized to VCP-normalized gephyrin signal intensity and expressed relative to VCP-normalized GABA_A_R subunit signal intensity in the total homogenate lane. We evaluated the expression of the target GABA_A_R subunits and, where applicable, calculated the ratio of individual isoforms to total subunit expression in each fraction lane. In addition, the ratios of α1:α2, β1:β2 and β2:β2 (total subunit and ratios to individual subunit isoforms) GABA_A_R subunit isoforms were assessed in each fraction between schizophrenia and comparison subjects.

Statistica (StatSoft, Tulsa, OK, USA) and Prism 6.0 (GraphPad Software, La Jolla, CA, USA) software were used for all the statistical analyses. Individual data points were excluded from statistical analysis if the protein expression of the target or normalizing factor was below the threshold for detection or greater than 4 s.d. from the mean. We first assessed all the dependent variables for normal distributions using the D'Agostino and Pearson omnibus normality test. For dependent variables that were not normally distributed, we used the Mann–Whitney *U*-test, and, for normally distributed dependent variables, we determined differences between groups using two-tailed Student's *t*-tests. To determine whether there were any significant associations between the dependent variables and potential covariates, we performed correlation analysis between the dependent measures and age, pH and postmortem interval, and found no significant correlations for any measures. There were no differences between diagnostic groups for age, pH or postmortem interval. Direct measures of GABA_A_R subunits in the total homogenates were compared to confirm our prior report of unchanged expression in the total STG homogenates in schizophrenia,^36^ as well as to validate their utility as normalizing factors for subunit expression by subject in each fraction. Direct measures of GABA_A_R subunits in the ER and SYN fractions were assessed between diagnostic groups to test specific hypotheses. Calculated measures of subunit ratios in the ER and SYN fraction were not based on independent hypotheses and were corrected for multiple testing using the Benjamini–Hochberg *q*-value,^73^ which controls for the false discovery rate. For multiple comparison tests, q*=0.05.

Although the male:female ratio differs between groups, *post hoc* two-way analysis of variance was performed for all the significant dependent measures and no sex effect was identified. In addition, *post hoc* Mann–Whitney *U*-tests between males and females within diagnostic groups were performed and no difference in expression within groups was identified (Supplementary Figure 1). *Post hoc* Mann–Whitney *U*-tests were also performed to assess differences between schizophrenia subjects ‘on' antipsychotic medication versus ‘off' medication for all the significant dependent measures. ‘Off' medication in this study is defined as >6 weeks of abstinence from antipsychotic medication before death. A medication effect was identified for the ratios of β1:β2_ALL_ and β1:β2_50 kDa_ in the SYN fraction (Supplementary Figure 2); no effect of neuroleptic treatment was discernible for any of the other significant variables. The majority of subjects in this study are Caucasian, with two Asian and one Hispanic subjects in the comparison group and three Black subjects and one subject of unknown race in the schizophrenia group; due to small group sizes, no meaningful *post hoc* statistical analyses of any effect of race on dependent measures were possible. For all the statistical analyses, *α*=0.05.

## Results

### The β2 GABA_A_R subunit, specifically the β2_50 kDa_ isoform, is increased in the ER fraction in schizophrenia

**Figure 2:**
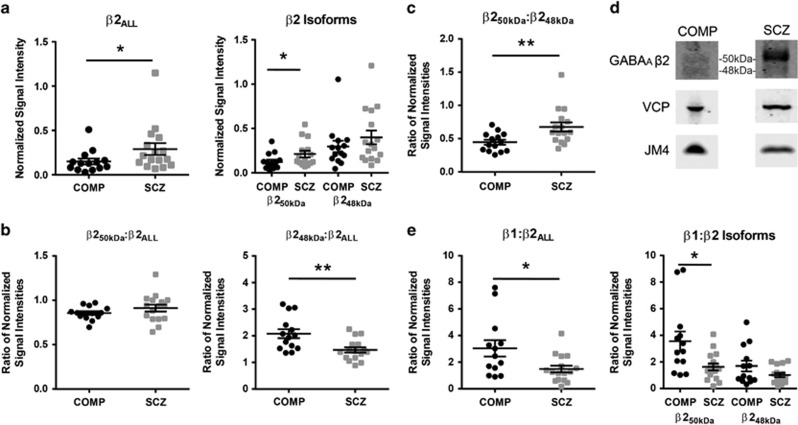
The β2 GABA_A_R subunit is abnormally expressed in an isoform-specific manner and the ratios of β1 and β2 subunit isoforms are altered in the ER in schizophrenia. Western blot analysis of total β2 GABA_A_R subunit (β2_ALL_) and individual β2 GABA_A_R subunit 50 kDa and 48 kDa isoforms (β2_50 kDa_ and β2_48 kDa_, respectively), the ratios of β2 GABA_A_R subunit isoforms to each other, and the ratio of β1:β2 GABA_A_R subunit and subunit isoform expression in the ER fraction in schizophrenia and comparison subjects. (**a**) ER fraction-normalized expression of β2_ALL_, and specifically the β2_50 kDa_ GABA_A_R subunit isoform, is increased in schizophrenia. (**b**) The ratio of β2_48 kDa_:β2_ALL_ GABA_A_R subunit fraction-normalized expression is decreased in the ER in schizophrenia. (**c**) The ratio of β2_50 kDa_:β2_48 kDa_ GABA_A_R subunit fraction-normalized signal intensities is increased in schizophrenia. (**d**) Representative images of western blots of the β2 GABA_A_R subunit, VCP and JM4 from the ER fraction from comparison and schizophrenia subjects with the β2_50 kDa_ and β2_48 kDa_ protein bands indicated. (**e**) The ratio of β1:β2_ALL_, and specifically the ratio of β1:β2_50 kDa_ GABA_A_R subunit expression is significantly less in the ER fraction in schizophrenia. Data are expressed as either the signal intensity of protein targets in the ER fraction normalized to VCP as a loading control and JM4 as an ER marker relative to the VCP-normalized signal intensity of the same target in the total homogenate, or expressed as a ratio of normalized signal intensities, for each data point with means±s.e.m. for each group indicated in **a**, **b**, **c** and **e**. *P<0.05, **P<0.01. COMP, comparison subject; ER, endoplasmic reticulum; GABA_A_R, γ-aminobutyric acid type A receptor; SCZ, schizophrenia; VCP, valosin-containing protein.

The β2 GABA_A_R subunit is visualized as multiple isoforms in a fraction-specific manner, with bands at ~52 and 50 kDa in the total homogenate; 50 and 48 kDa in the ER fraction (Figure 2d); and 52, 50 and 48 kDa in the SYN fraction. ER expression of all β2 isoforms (β2_ALL_) was 93% higher in schizophrenia (*U*(14,16)=61, *P*=0.03; Figure 2a, Table 2), and ER expression of the primary 50 kDa β2 isoform (β2_50 kDa_), which is seen in all the subcellular fractions, was 70% higher in the ER in schizophrenia (*U*(14,15)=59, *P*<0.05; Figure 2a, Table 2). There was no difference in the relative expression of the 48 kDa β2 isoform (β2_48 kDa_) in the ER (Figure 2a, Table 2) and no difference in the relative expression of β2_ALL,_ β2_50 kDa_, β2_48 kDa_ or 52 kDa β2 (β2_52 kDa_) GABA_A_R subunit isoforms in the total homogenate or SYN fractions between schizophrenia and comparison subjects (Table 2).

### The ratios of β2 GABA_A_R subunit isoforms are altered in the ER and SYN fractions in schizophrenia

**Figure 3:**
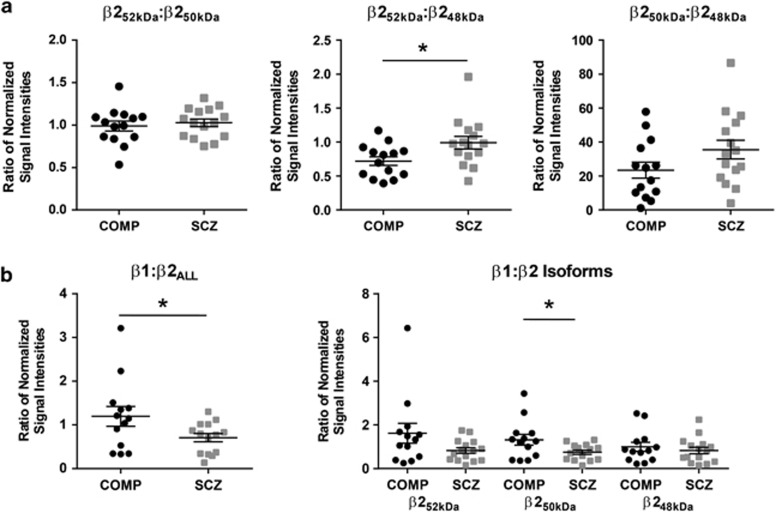
The ratio of β1:β2_ALL_, β1:β2_50 kDa_ and β2_52 kDa_:β2_48 kDa_ GABA_A_R subunit expression is increased in the SYN fraction in schizophrenia. Western blot analysis of the ratios of β1 and β2 GABA_A_R subunit isoform expression in the SYN fraction in schizophrenia and comparison subjects. (**a**) The ratio of β2_52 kDa_:β2_48 kDa_ GABA_A_R subunit expression is increased in schizophrenia, with no difference between groups for the ratio of β2_52 kDa_:β2_50 kDa_ or β2_50 kDa_:β2_48 kDa_ GABA_A_R subunit expression in the SYN fraction. (**b**) The ratio of β1:β2_ALL_ and β1:β2_50 kDa_ GABA_A_R subunit expression is decreased in the SYN fraction in schizophrenia. Data are expressed as a ratio of the signal intensity of protein targets in the SYN fraction normalized to VCP as a loading control and gephyrin as an inhibitory synaptic marker relative to the VCP-normalized signal intensity of the same targets in total homogenate for each subject; data are means±s.e.m. *P<0.05. COMP, comparison subject; GABA_A_R, γ-aminobutyric acid type A receptor; SCZ, schizophrenia; SYN, synapse; VCP, valosin-containing protein.

In the ER fraction, the ratio of β2_48 kDa_:β2_ALL_ GABA_A_R subunit expression is decreased in schizophrenia (*t*(28)=3.2, *P*<0.01, *q*<0.01; Figure 2b, Table 3) and is not different in the total homogenate or the SYN fraction (Table 3). The ratio of β2_50 kDa_:β2_48 kDa_ GABA_A_R subunit expression is increased in the ER in schizophrenia (*U*(14,16)=45, *P*<0.01, *q*=0.01; Figure 2c, Table 3) but unchanged in the SYN fraction (Figure 3a, Table 3). The ratio of β2_52 kDa_:β2_48 kDa_ GABA_A_R subunit expression is not different in total homogenate (Table 3), but is increased in the SYN fraction (*U*(14,15)=54, *P*=0.03, *q*<0.01; Figure 3a, Table 3). There is no difference in the ratio of β2_50 kDa_:β2_ALL_ GABA_A_R subunit expression between diagnostic groups in the ER (Figure 2b, Table 3), nor is there a difference in the total homogenate or SYN fractions for the ratios of β2_50 kDa_:β2_ALL_ or β2_52 kDa_:β2_50 kDa_ GABA_A_R subunit expression (Figure 3a, Table 3).

### The ratio of β1 to β2 GABA_A_R subunit expression is decreased in the ER and SYN fractions in schizophrenia

**Figure 4:**
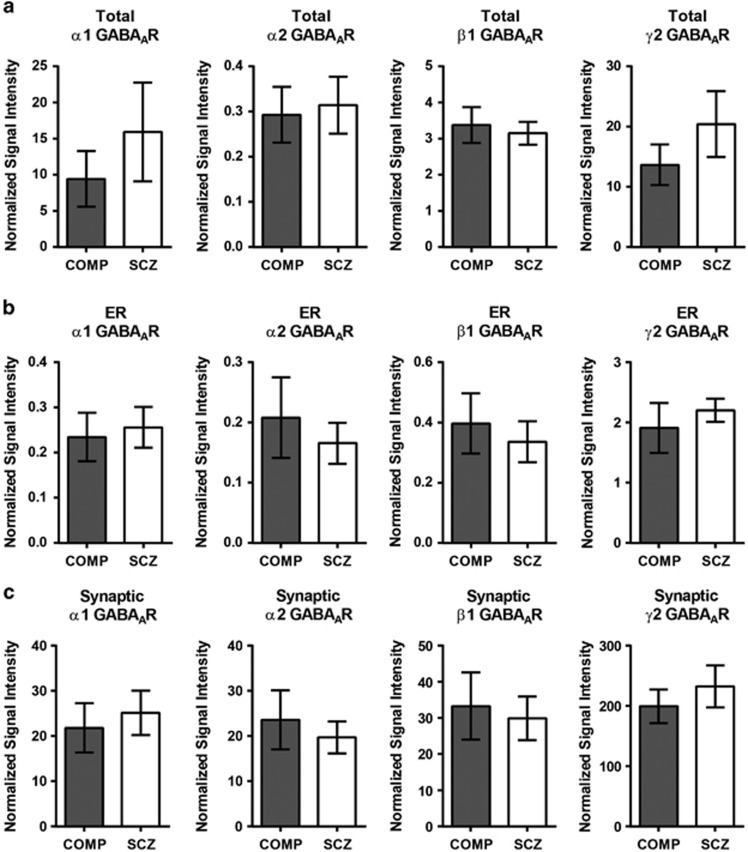
α1, α2, β1 and γ2 GABA_A_R subunit expression are not different between diagnostic groups in the total homogenate, ER or SYN fractions. Western blot analysis of α1, α2_ALL_, β1 and γ2 GABA_A_R subunit expression in schizophrenia and comparison subjects. There are no differences between diagnostic groups in the protein expression of α1, α2_ALL_, β1 or γ2 GABA_A_R subunits in (**a**) the total homogenates, (**b**) ER fractions or (**c**) the SYN fractions. Data are expressed as the mean signal intensity (±s.e.m.) of protein targets in the ER fraction normalized to VCP as a loading control, and JM4 as an ER marker or gephyrin as an inhibitory synapse marker, relative to the VCP-normalized signal intensity of the same target in the total homogenate. COMP, comparison subject; ER, endoplasmic reticulum; GABA_A_R, γ-aminobutyric acid type A receptor; SCZ, schizophrenia; SYN, synapse; VCP, valosin-containing protein.

There was no difference in the relative amount of the β1 GABA_A_R subunit expressed in the total, ER or SYN fractions between schizophrenia and comparison subjects (Figure 4, Table 2). However, the ratio of β1:β2 _ALL_ GABA_A_R subunit expression, while not different in total homogenate (Table 3), was significantly reduced in the ER (*U* (13,16)=53, *P*=0.03, *q*=0.03; Figure 2e, Table 3) and SYN fractions (*U* (13,14)=49, *P*=0.04; Figure 3b, Table 3) in schizophrenia. The ratio of β1:β2_50 kDa_ expression was also less in both the ER (*U* (13,16)=49, *P*=0.02, *q*=0.02; Figure 2e, Table 3) and SYN (*t* (25)=2.2, *P*=0.04; Figure 3b, Table 3) fractions in schizophrenia, with no difference between groups in total homogenate (Table 3). The ratios of β1:β2_52 kDa_ and β1:β2_48 kDa_ were unchanged in the total homogenate (Table 3) and SYN fractions (Figure 3b, Table 3) in schizophrenia and the ratio of β1:β2_48 kDa_ was also unchanged in ER (Figure 2a, Table 3). *Post hoc* analysis of medication status found the ratio of β1:β2_ALL_ in the SYN fraction decreased in schizophrenia subjects ‘off' medication relative to comparison subjects (*U* (3,13)=5, *P*=0.05) as well as a decrease of the β1:β2_ALL_ ratio in schizophrenia subjects ‘off' medication versus ‘on' medication (*U* (3,11)=3, *P*=0.04) in the SYN fraction (Supplementary Figure 2). Similarly, the β1:β2_50 kDa_ ratio was greater in comparison subjects than schizophrenia subjects ‘off' medication (*U* (3, 13)=2, *P*=0.01) and was greater in schizophrenia subjects ‘on' medication versus ‘off' medication (*U* (3,14)=0, *P*<0.01; Supplementary Figure 2).

### The β2_52 kDa_ GABA_A_R subunit isoform represents an *N*-glycosylated form of β2

**Figure 5:**
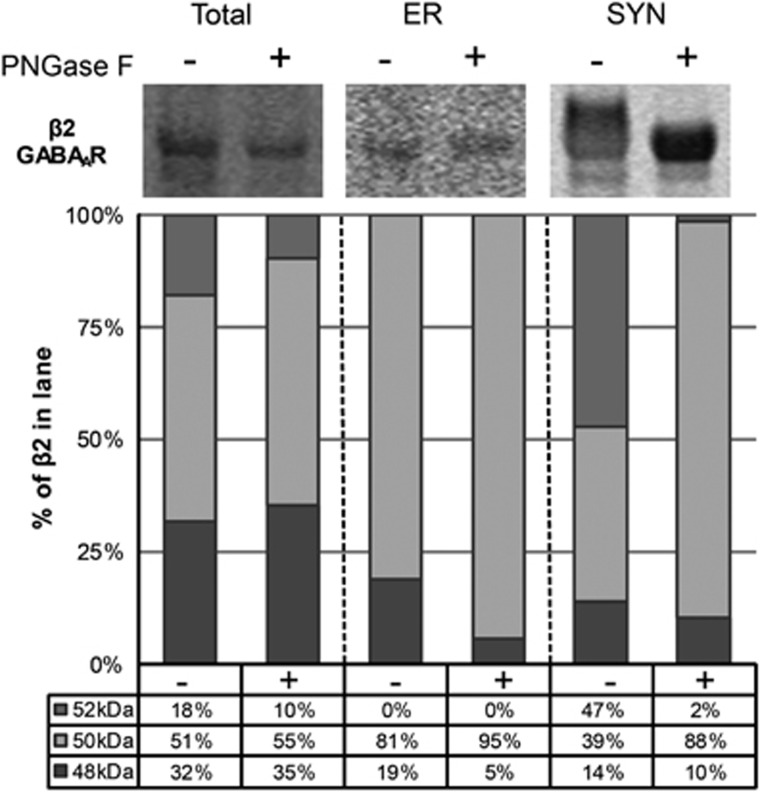
The β2_52 kDa_ GABA_A_R subunit isoform is *N*-glycosylated in postmortem human cortex. Representative images of western blots probed for the β2 GABA_A_R subunit in total homogenate, ER and SYN fractions with and without *N*-glycans cleaved by treatment with the deglycosylating enzyme PNGase F and corresponding graphs of β2 GABA_A_R subunit isoform protein expression as a percentage of total β2 GABA_A_R subunit in each lane. In brief, subcellular fractions generated from cortical homogenates were denatured and deglycosylated with PNGase F. Image Studio software was used to measure the signal intensity of protein bands at 52, 50 and 48 kDa in each lane. The signal intensity of each isoform was then divided by the sum of signal intensities for all the three isoforms to determine the percentage of total β2 GABA_A_R subunit expressed in the lane. After deglycosylation with PNGase F, the percentage of β2_52 kDa_ GABA_A_R is greatly reduced with a corresponding increase of β2_50 kDa_ GABA_A_R expressed in the SYN fraction. The calculated percentage of β2_48 kDa_ GABA_A_R in the ER fraction is also reduced after PNGase F treatment; however, this is likely an artifact due to the low signal intensity values for protein bands measured in those lanes. ER, endoplasmic reticulum; GABA_A_R, γ-aminobutyric acid type A receptor; PNGase F, peptide *N*-glycosidase F; SCZ, schizophrenia; SYN, synapse.

After cleavage of immature and mature *N*-glycans with PNGase F from subcellular fractions, the relative contribution of each β2 GABA_A_R subunit isoform to the total β2 GABA_A_R subunit protein expression was assessed in each fraction. The signal intensity of the β2_52 kDa_ GABA_A_R subunit isoform is greatly reduced and the signal intensity of the β2_50 kDa_ GABA_A_R subunit isoform exhibits a corresponding increase in signal intensity in the SYN fraction after deglycosylation (Figure 5). Although the calculated percentage of β2_48 kDa_ GABA_A_R in the ER fraction is also modestly reduced after PNGase F treatment, this is likely an artifact due to the low signal intensity values for protein bands measured in this assay from those lanes.

### Protein expression of α1, α2, β1 and γ2 GABA_A_R subunits is unchanged in the STG in schizophrenia

The α1 subunit appears as a single band at 52 kDa in the total homogenate, ER and SYN fractions. In the total homogenate and the SYN fraction, the α2 subunit appears as a doublet (α2_ALL_). The higher molecular mass band at ~51 kDa (α2_51 kDa_) is expressed discretely in the ER and the lower molecular mass band at 49 kDa (α2_49 kDa_) is expressed in the total homogenate and SYN fractions. The β1 GABA_A_R subunit appears as a doublet at ~50–52 kDa in the total homogenate, ER and SYN fractions. The γ2 GABA_A_R subunit is present in all the fractions and appears as a single band at ~51 kDa. There is no difference in the protein expression of the α1, β1 and γ2 GABA_A_R subunits between schizophrenia and comparison subjects in total homogenate, ER or SYN fractions (Figure 4, Table 2). There is also no significant difference between diagnostic groups in the expression of α2_ALL_ (Figure 4, Table 2), nor the α2_51 kDa_ or α2_49 kDa_ isoforms when assessed individually in any fraction (Table 2).

## Discussion

These data indicate abnormal subcellular expression of the β1 and β2 GABA_A_R subunits in the schizophrenia brain. We have previously reported that the 49 kDa β1 GABA_A_R subunit is more immaturely *N*-glycosylated and the total *N*-glycosylation of the β2 subunit is altered in schizophrenia, suggesting a mechanism underlying abnormal GABA_A_R subunit assembly, altered cell surface expression and trafficking disruptions in this illness.^36^ Although we did not find any abnormalities in β1 GABA_A_R subunit expression in subcellular compartments, we identified increased expression of β2_ALL_ driven by increased β2_50 kDa_ in the ER in schizophrenia.

To assess the relative abundance and localization of the β1 and β2 GABA_A_R subunits and β2 GABA_A_R subunit isoforms, we calculated the ratios of β1 and β2 isoforms in the ER and SYN fractions to assess potential differences in GABA_A_R subunit composition between schizophrenia and comparison subjects. In the ER fraction, we identified a significant decrease in the ratios of β1:β2_ALL_, β1:β2_50 kDa_ and β2_48 kDa_:β2_ALL_ and a significant increase in the ratio of β2_50 kDa_:β2_48 kDa_. Together, these data suggest a relative reduction in the expression of β1 GABA_A_R subunits, as well as a reduction of β2_48 kDa_ isoform expression in the ER in schizophrenia. In addition, these data suggest that the β2_50 kDa_ isoform is expressed more abundantly in the ER, which may reflect that this isoform is more likely to be incorporated into intact receptors trafficked to the cell membrane for expression at the synapse.

Consistent with our findings in the ER, the ratios of β1:β2_ALL_ and β1:β2_50 kDa_ GABA_A_R subunits were decreased in the SYN fraction, suggesting a relative increase of β2_50 kDa_ isoform expression and a relative decrease in β1 GABA_A_R subunit expression at the synapse in schizophrenia. We also determined that the ratio of β2_52 kDa_:β2_48 kDa_ GABA_A_R subunit isoforms is increased in the SYN fraction in schizophrenia, which we consider to be indicative of increased synaptic β2_52 kDa_ and decreased synaptic β2_48 kDa_. Because we could not measure the β2_52 kDa_ GABA_A_R isoform in the ER fraction, and the difference associated with β2_52 kDa_ is only apparent relative to β2_48 kDa_ isoform expression in the SYN fraction, it is unclear whether the β2_52 kDa_ isoform is specifically increased at the synapse; however, despite this confound, the altered ratios in the SYN fraction appear to be consistent with a relative increase of the β2_52 kDa_ and β2_50 kDa_ GABA_A_R subunit isoforms, and a relative decrease of β1 and β2_48 kDa_ GABA_A_R subunits incorporated into synaptic GABA_A_Rs. We have also shown that the β2_52 kDa_ GABA_A_R subunit in the SYN fraction likely represents an *N*-glycosylated form of β2 which, consistent with previous *in vitro* studies, suggests that *N*-glycosylated forms of the β2 GABA_A_R subunit may be preferentially incorporated into intact, synaptically expressed receptors. Interestingly, the decreased ratios of β1:β2_ALL_ and β1:β2_50 kDa_ in the SYN fraction in schizophrenia appear to be ameliorated by the effects of antipsychotic medications, with schizophrenia subjects ‘on' medication more closely resembling comparison subjects. This suggests that treatment with antipsychotic medication may result in increased expression of β1-containing GABA_A_Rs expressed at the synapse relative to those containing β2 subunits, possibly by inhibiting incorporation of the β2_50 kDa_ subunit into synaptically targeted receptors.

In humans, the β2 GABA_A_R subunit protein is expressed as four isoforms (β2_L_, β2_S_, β2_S1_ and β2_S2_) that are the result of mRNA splice variants which can be regulated by epigenetic modifications at key neurodevelopmental time points.^74, 75, 76, 77^ The β2_L_ and β2_S_ isoforms are distinguished by the inclusion of exon 10 in the β2_L_ isoform, to produce subunits with predicted molecular masses of 60 kDa and 54 kDa, respectively.^75, 76^ The β2_S1_ and β2_S2_ isoforms were subsequently identified with predicted molecular masses of 36 kDa and 42 kDa, respectively, and are differentiated from the β2_L_ and β2_S_ isoforms by the exclusion of the fourth transmembrane domain of the subunit.^75^ On the basis of the predicted molecular masses of β2 GABA_A_R subunit isoforms, we propose that the antibody used in this study labeled posttranslationally modified and unmodified forms of the β2_S_ and/or β2_S2_ isoforms.

In genetic studies, chromosome 5q34, where the GABRB2 gene is located, has been identified as a region of interest for schizophrenia-related risk alleles.^34, 78, 79^ In addition, schizophrenia has been associated with a recombination hotspot^80^ and there is evidence for multiple schizophrenia-associated single-nucleotide polymorphisms in both coding and non-coding regions of the GABRB2 gene.^74, 76, 80, 81, 82^ Some GABRB2 single-nucleotide polymorphism haplotypes appear to be subject to regulation by parental imprinting or other epigenetic modifications, which may explain inconsistencies in previous reports examining the role of chromosome 5 in schizophrenia susceptibility risk in different patient populations.^74, 79, 83^ The variability of GABRB2 mRNA splice variant expression and corresponding alterations in β2 GABA_A_R subunit protein isoforms expressed as a result of epigenetic or neurodevelopmental changes supports the two-hit model of schizophrenia,^84^ which posits that genetic predisposition to the disorder in combination with some environmental factor(s) contributes to the onset of psychosis and the conversion from prodromal to symptomatic patient phenotypes.

It has been established that the subunit composition of heteropentameric GABA_A_Rs affects the signaling properties of the receptor,^25, 26, 52, 85, 86, 87, 88, 89, 90, 91, 92^ and it has more recently been shown that the specific β2 GABA_A_R subunit isoform incorporated into the intact receptor has a significant role in the functional and electrophysiological properties of GABA_A_Rs.^75, 82, 89^ In addition to isoform-specific differences in GABAergic signaling, posttranslational modifications of the β2 GABA_A_R subunit are known to affect heteropentamer assembly, receptor trafficking, cell surface expression, membrane stability, ligand-binding affinity, channel gating properties and receptor kinetics.^91, 93^ *N*-glycosylation-deficient β2 GABA_A_R subunits expressed in binary α1β2 GABA_A_Rs in the plasma membrane *in vitro* display reduced current amplitude and decreased long single-channel openings; *N*-glycosylation of β2 GABA_A_R at N104 has been shown to affect heteropentamer assembly; and proper immature *N*-glycosylation at N173 affects the stability of individual β2 GABA_A_R subunits in the ER,^93^ which together illustrate that early protein processing can substantially affect not only the composition and surface expression of specific β2 GABA_A_R subunit isoforms, but also the signaling properties of intact GABA_A_Rs.

On the basis of prior findings that the α2 GABA_A_R subunit is expressed more highly in intact GABA_A_Rs in axosomatic synapses of pyramidal neurons in the dorsolateral prefrontal cortex in schizophrenia,^60^ in conjunction with our prior report demonstrating a smaller immature *N*-glycan attached to the α1 GABA_A_R subunit in schizophrenia,^36^ we anticipated finding increased abundance of α1 GABA_A_R subunits in the ER fraction, consistent with retention of this subunit in the calnexin–calreticulin protein-folding cycle, and a decreased ratio of α1:α2 GABA_A_R subunits in both the ER and SYN fractions. Although we found no difference in the abundance of the α1 or α2 GABA_A_R subunits, nor a change in the ratio of α1:α2 GABA_A_R subunits in the ER or SYN fractions in schizophrenia versus comparison subjects, this can be reconciled with the earlier findings. It has been shown *in vitro* that only 25% of translated subunits are assembled into intact GABA_A_Rs, which are then trafficked to the cell membrane;^53, 94^ and although our previous *N*-glycosylation findings indicate possible α1 GABA_A_R subunit retention in the ER, it is also possible that aberrantly *N*-glycosylated α1 GABA_A_R subunits may be rapidly expelled from the ER and undergo ER-associated degradation via the ubiquitin–proteasome system instead of remaining sequestered in the ER.^42, 53, 54, 55, 95^

Because α1 and β2 GABA_A_R subunits preferentially co-assemble in intact receptors, and our data suggest relatively more β2 GABA_A_R expression in the ER and SYN fractions, another possible explanation may be that the increased expression of β2 versus β1 GABA_A_R subunits in schizophrenia facilitates α1 versus α2 assembly into intact, synaptically targeted GABA_A_Rs. In addition, because we used specific biochemical methods to isolate a synapse-enriched fraction from cortical homogenate, the SYN fraction is enriched for a combination of excitatory and inhibitory synapses, as well as a combination of axosomatic and dendritic synapses and, as such, we may be unable to identify alterations that are specific to inhibitory axosomatic synapses on pyramidal neurons. The possibility that altered ratios of α1:α2 GABA_A_R subunits may be masked when measured in our assays, or that α-subunit-specific alterations may be more readily evident in the other cortical areas, such as the dorsolateral prefrontal cortex, cannot be ruled out.

We measured the expression of γ2 GABA_A_R subunits in the fractions as an indirect measure of intact GABA_A_R localization, owing to the role of the γ2 subunit in synaptic targeting via its interaction with GABA_A_R-associated protein, GABARAP, an essential component of the GABA_A_R trafficking machinery.^52, 53, 55, 56, 57^ We found no difference in γ2 GABA_A_R subunit expression in the ER or SYN fractions between schizophrenia and comparison subjects. This suggests that intact synaptically targeted heteropentameric GABA_A_Rs are being assembled in the ER and localized to the synapse, but does not exclude the possibility that other GABA_A_R subunits or specific subunit isoforms incorporated into γ2-containing GABA_A_R s may be altered in schizophrenia.

As with all the postmortem studies, there are several limitations to this work. As mentioned previously, the diagnostic groups were not equally matched for sex. Although we did not identify any sex effects for any significant dependent measure (Supplementary Figure 1), the relatively small sample size may not be sufficient to reliably identify sex-specific abnormalities of GABA_A_R subunit expression and localization in schizophrenia. In addition, the age range of subjects in this study was 53–97 years at the time of death; thus, these findings may not be generalizable to younger patients in the earlier stages of the disorder. *Post hoc* statistical analyses were performed in an effort to control for these limitations.

Given our previous report of increased immature *N*-glycosylation of the β1_49 kDa_ GABA_A_R subunit and altered total *N*-glycosylation of the β2 GABA_A_R subunit, our current data indicating increased β2_50 kDa_ and decreased β1 and β2_48 kDa_ GABA_A_R subunits in both the ER and SYN fractions and increased β2_52 kDa_ in the SYN fraction in schizophrenia provide evidence that proper ER processing and synaptic targeting of β1- and β2-containing GABA_A_Rs are affected by *N*-glycosylation abnormalities in schizophrenia. Our current data suggest that there is an increase of *N*-glycosylated β2_52 kDa_ GABA_A_R subunits expressed synaptically in the STG in schizophrenia. The disparate expression of β2 subunit isoforms at the synapse suggests a GABA_A_R subunit-mediated postsynaptic abnormality in GABAergic signaling in schizophrenia and, as such, could potentially be a target for pharmacological intervention. The subunit composition of GABA_A_Rs is disrupted in multiple brain regions in schizophrenia, and although prior studies have highlighted alterations in membrane expression of the α1 and α2 GABA_A_R subunits, further investigation of the functional consequences of aberrant β1 and β2 GABA_A_R subunit isoform membrane expression may provide additional insight into the etiology of GABAergic signaling deficits in schizophrenia.

## Figures and Tables

**Table 1:** Summary of subject demographics

	*Comparison*	*Schizophrenia*
*n*	14	16
Age	79.4±9.3	75.8±11.9
Sex	4 M/10 F	11 M/5 F
PMI (h)	10.0±7.3	11.4±4.4
Tissue pH	6.3±0.2	6.4±0.3
On/off Rx	0/14	11/5

Abbreviations: F, female; M, male; PMI, postmortem interval; Rx, antipsychotic medication.

Values are expressed as means±s.d. Off Rx indicates patients that had not received antipsychotic medications for 6 weeks or more at the time of death.

**Table 2:** α1, α2, β1 and γ2 GABA_A_ receptor subunit protein expression is unchanged in the total homogenate, ER and synapse-enriched fractions of the STG in schizophrenia

*GABA*_*A*_*R subunit*	*Comparison*	*Schizophrenia*	*Test statistic (d.f.)*	P*-value*
*Total*				
α1	9.42±13.88	15.91±26.47	*U* (13,15)=95	
α2_ALL_	0.29±0.23	0.31±0.25	*U* (14,16)=105	
α2_51 kDa_	0.13±0.12	0.12±0.08	*U* (13,15)=107	
α2_49 kDa_	0.16±0.13	0.19±0.19	*U* (14,16)=104	
β1	3.37±1.86	3.15±1.23	*t* (27)=0.39	
β2_ALL_	0.43±0.29	0.36±0.25	*U* (14,16)=95	
β2_52 kDa_	0.15±0.10	0.11±0.05	*U* (14,16)=90	
β2_50 kDa_	0.25±0.20	0.20±0.12	*U* (14,16)=96	
β2_48 kDa_	0.11±0.07	0.11±0.07	*t* (28)<0.01	
γ2	13.64±12.19	20.42±20.43	*U* (13,16)=67	
				
*ER*				
α1	0.23±0.19	0.26±0.18	*U* (12,15)=80	
α2_ALL_	0.21±0.24	0.17±0.14	*U* (13,16)=99	
α2_51_	0.29±0.32	0.25±0.16	*U* (13,16)=91	
β1	0.40±0.36	0.34±0.27	*U* (13,16)=99	
β2_ALL_	**0.15±0.12**	**0.27±0.27**	***U*** **(14,16)=61**	**0.03**
β2_50 kDa_	**0.12±0.09**	**0.21±0.15**	***U*** **(14,15)=59**	**<0.05**
β2_48 kDa_	0.30±0.24	0.40±0.31	*U* (14,16)=93	
γ2	1.91±1.49	2.20±0.77	*t* (25)=1.03	
				
*Synaptic*				
α1	21.83±19.70	25.19±18.32	*U* (13,14)=75	
α2_ALL_	23.65±23.55	19.71±13.29	*t* (27)=0.54	
α2_51_	50.41±31.80	49.88±35.37	*U* (13,15)=82	
α2_49_	28.57±31.81	23.06±19.54	*U* (13,14)=80	
β1	33.31±33.48	29.93±23.39	*U* (13, 15)=94	
β2_ALL_	31.00±23.01	39.87±25.53	*t* (27)=0.98	
β2_52 kDa_	22.13±11.82	39.55±25.90	*U* (13,15)=64	
β2_50 kDa_	28.09±21.46	37.39±21.84	*U* (14,15)=74	
β2_48 kDa_	39.34±31.59	45.62±35.16	*U* (14,15)=96	
γ2	199.80±96.41	232.70±120.50	*t* (22)=0.74	

Abbreviations: ER, endoplasmic reticulum; GABA_A_R, γ-aminobutyric acid type A receptor; STG, superior temporal gyrus.

Values are reported as means±s.d. For normally distributed dependent measures, data were analyzed using Student's *t*-tests; for dependent measures that were not normally distributed, data were analyzed using the Mann–Whitney *U*-test. Test statistics which met the threshold for significance (*α*=0.05) are listed in bold.

**Table 3:** Ratios of GABA_A_R subunit isoforms are altered in schizophrenia in the ER and synaptic fractions

*GABA*_*A*_*R subunit isoform ratios*	*Comparison*	*Schizophrenia*	*Test statistic (d.f.)*	P*-value*	q*-value*
*Total*					
α1:α2	50.41±31.80	49.88±35.37	*U* (12,12)=71		
β1:β2_ALL_	9.33±4.89	9.20±4.83	*U* (14,14)=96		
β1:β2_52 kDa_	24.67±14.42	27.46±10.69	*U* (13,14)=63		
β1:β2_50 kDa_	15.16±5.97	17.36±9.04	*U* (14,14)=91		
β1:β2_48 kDa_	31.94±14.35	33.11±16.75	*t* (25)=0.19		
β2_52 kDa_:β2_ALL_	0.36±0.08	0.35±0.12	*t* (28)=0.26		
β2_50 kDa_:β2_ALL_	0.60±0.12	0.60±0.19	*U* (14,16)=99		
β2_48 kDa_:β2_ALL_	0.26±0.04	0.30±0.12	*U* (14,16)=93		
β2_52 kDa:_β2_50 kDa_	0.62±0.21	0.60±0.21	*t* (28)=0.25		
β2_52 kDa:_β2_48 kDa_	1.39±0.38	1.20±0.40	*t* (28)=1.35		
β2_50 kDa:_β2_48 kDa_	2.39±0.73	2.12±0.86	*U* (14,16)=81		
					
*ER*					
α1:α2	1.40±0.97	1.15±0.65	*t* (26)=0.81		
β1:β2_ALL_	**3.04±2.21**	**1.48±1.02**	***U*** **(13,16)=53**	**0.03**	**0.03**
β1:β2_50 kDa_	**3.56±2.60**	**1.63±1.04**	***t*** **(27)=2.72**	**0.01**	**0.02**
β1:β2_48 kDa_	1.69±1.45	1.01±0.64	*t* (27)=1.70		
β2_50 kDa_:β2_ALL_	0.86±0.08	0.91±0.16	*t* (28)=1.23		
β2_48 kDa_:β2_ALL_	**2.08±0.63**	**1.47±0.40**	***t*** **(28)=3.21**	**<0.01**	**<0.01**
β2_50 kDa:_β2_48 kDa_	**0.45±0.14**	**0.68±0.27**	***U*** **(14,16)=45**	**<0.01**	**0.01**
					
*Synaptic*					
α1:α2	1.07±0.88	1.01±0.72	*U* (13,13)=84		
β1:β2_ALL_	**1.19±0.82**	**0.71±0.36**	***U*** **(13,14)=49**	**0.04**	0.01
β1:β2_52 kDa_	3.04±2.21	1.48±1.02	*U* (13,15)=66		
β1:β2_50 kDa_	**3.04±2.21**	**1.48±1.02**	***t*** **(25)=2.20**	**0.04**	0.01
β1:β2_48 kDa_	3.04±2.21	1.48±1.02	*t* (26)=0.67		
β2_52 kDa_:β2_ALL_	0.88±0.19	0.95±0.18	*t* (26)=0.99		
β2_50 kDa_:β2_ALL_	0.90±0.06	0.94±0.13	*U* (14,14)=80		
β2_48 kDa_:β2_ALL_	1.31±0.38	1.19±0.59	*U* (14,15)=69		
β2_52 kDa:_β2_50 kDa_	0.99±0.22	1.03±0.17	*t* (27)=0.52		
β2_52 kDa:_β2_48 kDa_	**0.72±0.24**	**0.99±0.36**	***U*** **(14, 15)=54**	**0.03**	<0.01
β2_50 kDa:_β2_48 kDa_	23.46±17.42	35.56±21.43	*t* (27)=1.66		

Abbreviations: ER, endoplasmic reticulum; GABA_A_R, γ-aminobutyric acid type A receptor.

Values are reported as means±s.d. For the normally distributed dependent measures, data were analyzed using Student's *t*-tests; for the dependent measures that were not normally distributed, data were analyzed using the Mann–Whitney *U*-test. Test statistics which met the threshold for significance (*α*=0.05, q*=0.05) are listed in bold.
