# Supplementary figures and images for: Abnormal subcellular localization of GABAA receptor subunits in schizophrenia brain
Source: Transl Psychiatry. 2015 Aug 4;5(8):e612–. doi: 10.1038/tp.2015.102 (PMC4564557; doi:10.1038/tp.2015.102)

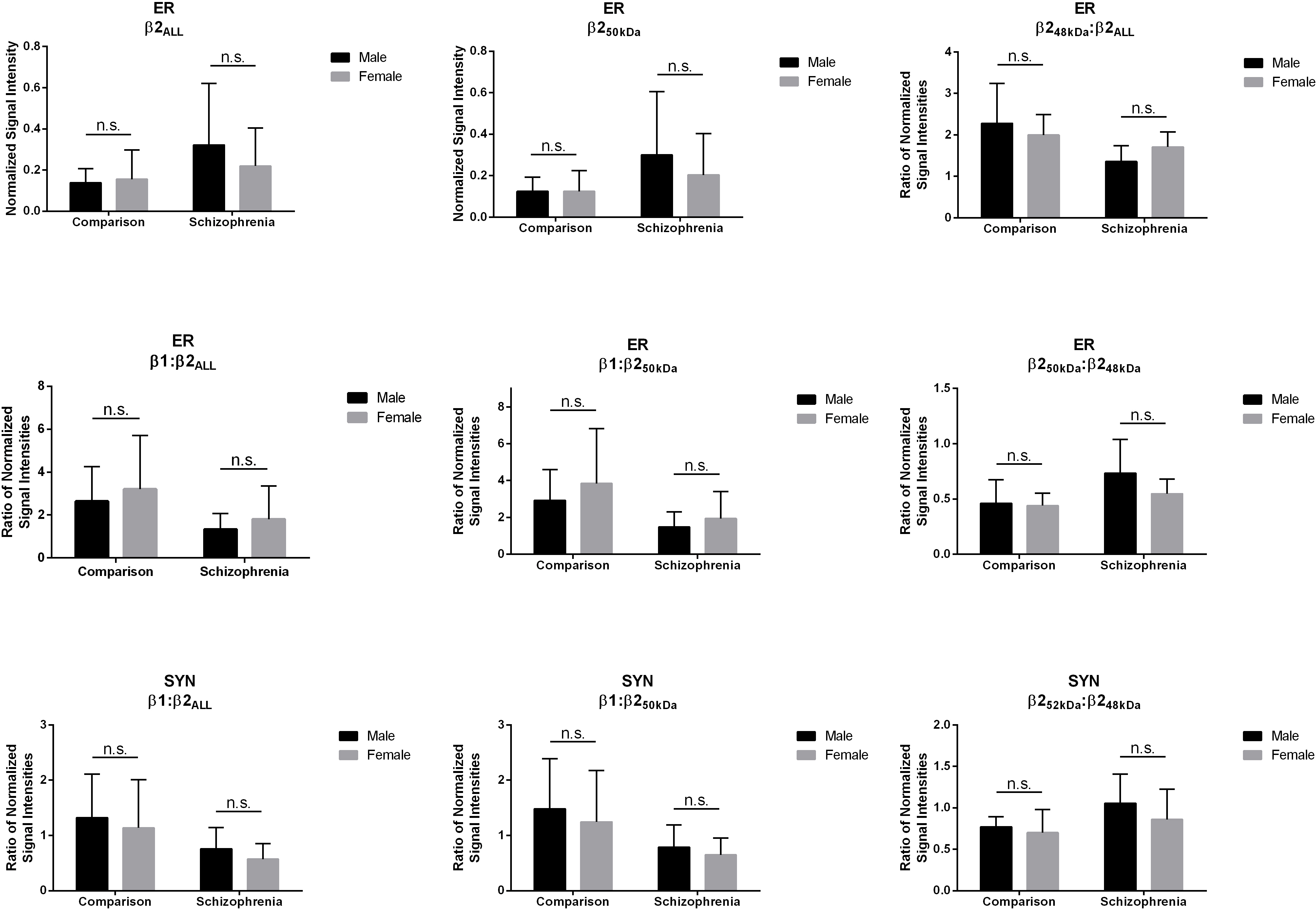

Supplement: Supplementary Figure 1 [file tp2015102x1.tif]

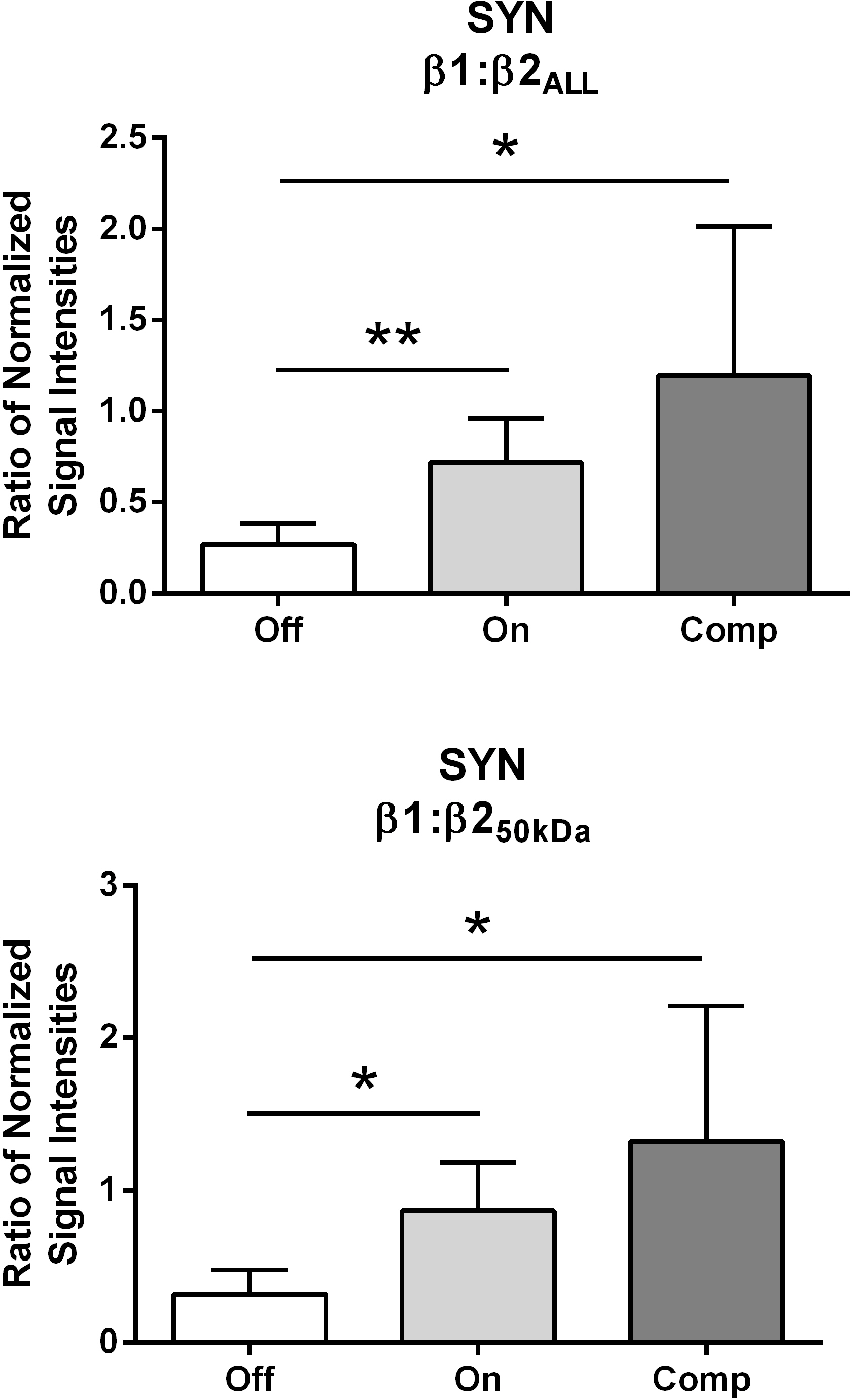

Supplement: Supplementary Figure 2 [file tp2015102x2.tif]
