# Supplementary material for: Abnormal subcellular localization of GABAA receptor subunits in schizophrenia brain
Source: Transl Psychiatry. 2015 Aug 4;5(8):e612–. doi: 10.1038/tp.2015.102 (PMC4564557; doi:10.1038/tp.2015.102)
Supplement: Supplementary Information [file tp2015102x3.doc]

**Supplemental Figure 1**

**The expression of β2_All_ and β2_50kDa_ and the ratios of β2_48kDa_:β2_All_, β2_50kDa_:β2_48kDa_, β1:β2_All_, and β1:β2_50kDa_ in the ER fraction and β1:β2_All_ and β1:β2_50kDa_ in the SYN fraction are not different between males and females within diagnostic groups.** Western blot analyses of β2_All_, β2_50kDa_, β2_48kDa_:β2_All_, β2_50kDa_:β2_48kDa_, β1:β2_All_, and β1:β2_50kDa_ in the ER fraction and β1:β2_All_, β1:β2_50kDa_, and β2_52kDa_:β2_48kDa_ in the SYN fraction between males and females within diagnostic groups. There is no difference in the protein expression of any significant measures or calculated ratios between males and females within a diagnostic group (schizophrenia or comparison). Data are expressed as means (± SD).

**Supplemental Figure 2**

**Decreased expression of β1:β2_All_ and β1:β2_50kDa_ in the SYN fraction is driven by decreased expression in schizophrenia subjects “off” medication.** Western blot analyses of β1:β2_All_ and β1:β2_50kDa_ in the SYN fraction between schizophrenia subjects “off” medication, schizophrenia subjects “on” medication, and comparison subjects in the SYN fraction. The ratios of β1:β2_All_ and β1:β2_50kDa_ are decreased in schizophrenia subjects “off” medication versus schizophrenia subjects “on” medication, as well as decreased between schizophrenia subjects “off” medication and non-psychiatrically ill comparison subjects. The ratios of β1:β2_All_ and β1:β2_50kDa_ between schizophrenia subjects “on” medication and comparison subjects are not significantly different. “Off” medication indicates schizophrenia subjects that had been abstinent from antipsychotic medications for greater than 6 weeks prior to time of death. Data are expressed as mean (± SD). *p < 0.05, **p < 0.01.
